# Ontogenesis of the Gut Microbiota Composition in Healthy, Full-Term, Vaginally Born and Breast-Fed Infants over the First 3 Years of Life: A Quantitative Bird’s-Eye View

**DOI:** 10.3389/fmicb.2017.01388

**Published:** 2017-07-21

**Authors:** Ravinder Nagpal, Hirokazu Tsuji, Takuya Takahashi, Koji Nomoto, Kazunari Kawashima, Satoru Nagata, Yuichiro Yamashiro

**Affiliations:** ^1^Probiotics Research Laboratory, Juntendo University Graduate School of Medicine Tokyo, Japan; ^2^Yakult Central Institute Kunitachi, Japan; ^3^Gonohashi Obstetrics and Gynecology Hospital Koto, Japan; ^4^Department of Pediatrics, School of Medicine, Tokyo Women’s Medical University Shinjuku, Japan

**Keywords:** bacterial quantification, gut bacterial communities, gut microbiome, intestinal microbiota development, RT-qPCR

## Abstract

Early-life intestinal microbiota development is crucial for host’s long-term health and is influenced by many factors including gestational age, birth and feeding modes, birth environment, ethnic/geographical background, etc. However, ‘quantitative’ data on the actual population levels of gut bacterial communities when these influences are controlled for is relatively rare. Herein, we demonstrate a quantitative perspective of microbiota development in natural and healthy milieus, i.e., in healthy, full-term, vaginally born and breast-fed infants (*n* = 19) born at same clinic. Fecal microbiota at age 1 and 7 days, 1, 3, and 6 months and 3 years is quantified using highly sensitive reverse-transcription-quantitative-PCR assays targeting bacterial rRNA molecules. At day 1, we detect one or more bacteria in all (100%) of the babies, wherein the microbiota is composed mainly of enterobacteria (35%), *Bacteroides fragilis* group (23%), enterococci (18%), staphylococci (13%), and bifidobacteria (9%). Altogether, facultative anaerobes predominate during first few weeks whereafter obligate anaerobes including bifidobacteria, *B. fragilis* group, *Clostridium coccoides* group, and *Clostridium leptum* subgroup gradually start prevailing. At 3 years, the composition is represented almost entirely (99%) by obligate anaerobes including *C. leptum* subgroup (34%), bifidobacteria (22%), *B. fragilis* group (21%), *C. coccoides* group (17%), *Atopobium* cluster (4%), and *Prevotella* (1%). The overall obligate/facultative proportion is 32/68, 37/63, 54/46, 70/30, 64/36, and 99/1% at 1 and 7 days, 1, 3, and 6 months and 3 years, respectively. However, interestingly, considerable individual-specific variations in the obligate/facultative ratios as well as in the proportions of Firmicutes, Bacteroides, Actinobacteria, and Proteobacteria communities are seen among these babies. This disparity even within this highly homogenous cohort manifests the magnitude of diverse patterns of gut microbiota configuration and hence underpins the importance of considering not only the gestational age, birth, and feeding modes, and ethnic/geographical background but also other potential outstanding factors when investigating the elements shaping the early microbiota development. In summary, the data demonstrate a quantitative bird’s-eye view of the ontogenesis of early-life gut microbiota in typically natural and healthy milieus and should be informative and facilitative for future studies exploring various aspects of the human gut microbiota.

## Introduction

The past decade has been remarkable in revealing the fundamental role of human gut microbiota in various aspects of host health and disease. It is now well established that the microbial colonization in human gut starts immediately at birth (Penders et al., 2006; Dominguez-Bello et al., 2010; Tsuji et al., 2012; Backhed et al., 2015; Bokulich et al., 2016; Nagpal et al., 2016) or perhaps even before that, i.e., *in utero* (Aagaard et al., 2014; Collado et al., 2016; Chu et al., 2017); and the magnitude and array of this early-life microbiota acquisition may affect several aspects of the newborn’s long-term health (Nagpal and Yamashiro, 2015; Tamburini et al., 2016). However, majority of the available knowledge has been acquired using bacterial 16S rRNA gene-based sequencing methods which, while being remarkably elaborative and comprehensive, are largely qualitative and may not provide precise data on fecal bacterial counts. In addition, the data acquired from DNA-based PCR or sequencing methods may also include unsought DNA entities (e.g., DNA of dead bacteria) and hence may not discriminate between viable and dead bacteria. As a result, quantitative longitudinal data on the actual population levels (i.e., the bacterial count) of various important bacterial clades dwelling in the infant gut during early life remains limited and disparate. Undeniably, even in this golden age of revolutionary sequence tools, the quantitative data denoting the ‘gold standard’ viable bacterial count still remains of indispensable worth. In this context, we have previously established a novel and sensitive analytical system for human intestinal microbiota analysis based on reverse-transcription-quantitative-PCR (RT-qPCR) assays targeting bacterial 16S rRNA ‘molecules,’ wherein we had methodologically validated that this RT-qPCR approach is relatively highly sensitive (detection limit: 10^3^–10^4^ bacterial cells/g feces; approximately 100- to 1000-fold higher than that of other molecular methods including qPCR and t-RFLP) and provides fecal bacterial enumeration comparable to the viable cell counts as enumerated by culturing and fluorescent *in situ* hybridization methods (Matsuda et al., 2007, 2009; Kubota et al., 2010; Kurakawa et al., 2015).

Utilizing this sensitive quantitative approach in longitudinal studies to examine the development of early life gut microbiota composition in a cohort of more than 150 Japanese infants, we have recently demonstrated that the mode of delivery (vaginal vs. cesarean birth) and the feeding type (breast-milk vs. formula-feed) have a strong influence on the intestinal carriage of various important bacterial clades and that the effects of these influences on gut microbiota start building up as early as the first day or week of life (Tsuji et al., 2012; Nagpal et al., 2016). Hence, these findings as well as the similar results from several other recent studies (Dominguez-Bello et al., 2010; Backhed et al., 2015; Dogra et al., 2015; Bokulich et al., 2016) instigated us to investigate afresh – and with a different perspective – the data of early life microbiota development when these influences are controlled for. Therefore, in this addendum, with an aim to manifest a quantitative bird’s-eye view of the ontogenesis of early gut microbiota configuration in natural and healthy milieus, we demonstrate the fecal microbiota composition in a selected cohort of 19 healthy, full-term, vaginally born and exclusively breast-fed infants prospectively from birth to 3 years of age. Unlike previous reports wherein we presented the data largely in the form of bacterial count and prevalence (Tsuji et al., 2012; Nagpal et al., 2016), this addendum focuses mainly on the relative proportions of various important gut bacterial groups and the ratios between obligate and facultative anaerobes as well as between Firmicutes, Bacteroides, Actinobacteria, and Proteobacteria, thereby outlining a characteristic profile of the early-life gut microbiota structure in a ‘healthy’ infant cohort that may be reflective of characteristics common to the present-day healthy Japanese infant population.

## Materials and Methods

The study includes 19 healthy Japanese infants enrolled at the Gonohashi Obstetrics and Gynecology Hospital, Tokyo. As mentioned beforehand, these infants are part of a large cohort of over 150 Japanese babies wherein fecal carriage of various gut microbes during the first 3 years of age has previously been reported (Nagpal et al., 2016). The general information about these infants is provided in **Table 1**, and the detailed information about the cohort can be found elsewhere (Nagpal et al., 2016). Briefly, all these 19 babies were full-term and vaginally born and remained exclusively breast-fed until 3 to 6 months of age. All the babies as well as their mothers remained apparently healthy with no indication of any major illness or any considerable exposure to antibiotics/drugs during the study period. The study design was approved by the ethical committees of the Juntendo University and the Yakult Central Institute, Tokyo. In accordance with the Declaration of Helsinki, prior written informed consent was obtained from all the parents or legal representatives.

**Table 1:** General characteristics of the 19 infants enrolled in the study.

Characteristics	Age					
	1 day	7 days	1 month	3 months	6 months	3 years
Girl : Boy	10 : 9			–			
Days (Avg. ± SD) spent in the hospital post-birth	4.0 ± 0.0			–			
Bodyweight, kg (Mean ± SD)	2.9 ± 0.2	2.9 ± 0.3	3.8 ± 0.5	5.7 ± 0.6	7.2 ± 0.7	13.2 ± 1.3
Exclusively breast-fed	19	19	19	19	8	–
Exclusively formula-fed	0	0	0	0	0	–
Mixed-fed	0	0	0	0	11	–
First exposure to formula-feed	0	0	0	0	11	–
Antibiotic exposure (baby)	0	0	0	0	0	0
Antibiotic exposure (mother)	0	1	0	0	0	0

Details of sample collection procedure, RNA extraction, RT-qPCR reactions, and primer specifics have been provided elsewhere (Nagpal et al., 2016). Briefly, to obtain an accurate description of the population levels of specific gut bacterial groups and to precisely enumerate changes in their fecal carriage, we used a highly sensitive analytical approach based on RT-qPCR assays targeting bacterial 16S rRNA molecules (Matsuda et al., 2009; Nagpal et al., 2016). As specified elsewhere (Nagpal et al., 2016), the minimum detection limit of these assays ranged from 10^3^ to 10^4^ cells/g feces. Using this analytical approach, we aimed to cover a wide-range of the human gut microbiota by targeting a wide array of anaerobic, facultative anaerobic, Gram-positive, Gram-negative, predominant, subdominant, indigenous pathobiont, and opportunistic pathogenic bacterial clades etc. that are frequently and prevalently associated with human gut microbiota. We herein quantified 11 different major gut bacterial groups including *Clostridium coccoides* group, *Clostridium leptum* subgroup, *Bacteroides fragilis* group, *Prevotella*, *Bifidobacterium*, *Atopobium* cluster, *Lactobacillus*, Enterobacteriaceae, *Enterococcus*, *Staphylococcus*, and *Clostridium perfringens* in the fecal specimens of these infants at six time-points viz. age 1 and 7 days, 1, 3, and 6 months, and 3 years. The bacterial counts enumerated by RT-qPCR assays are expressed as log_10_ cells per gram of feces (mean ± standard deviation), and the carriage rate or prevalence of colonization is expressed as the percentage of infants in which the specific bacterium was detected. The total bacterial count was estimated as the sum of the counts of these 11 bacterial clades. The count of genus *Bifidobacterium* was estimated as the sum of the counts of one group and seven species viz. *B. catenulatum* group (including *B. catenulatum* and *B. pseudocatenulatum*), *B. longum*, *B. infantis*, *B. bifidum*, *B. breve*, *B. adolescentis*, *B. angulatum*, and *B. dentium*. The count of genus *Lactobacillus* was estimated as the sum of the counts of eight subgroups/species, i.e., *L. gasseri* subgroup, *L. casei* subgroup, *L. plantarum* subgroup, *L. reuteri* subgroup, *L. ruminis* subgroup, *L. sakei* subgroup, *L. brevis*, and *L. fermentum*.

The proportions of each bacterial group were calculated with regard to the total bacterial count. Normalization was done by dividing the values of each bacterial group by the value obtained for the ‘total bacterial count.’ The proportions of obligate and facultative anaerobes were estimated by the sum of the corresponding bacterial groups (*C. coccoides* group, *C. leptum* subgroup, *B. fragilis* group, *Bifidobacterium*, *Atopobium* cluster, *Prevotella* and *C. perfringens* for obligate anaerobes; and Enterobacteriaceae, *Enterococcus*, *Lactobacillus* and *Staphylococcus* for facultative anaerobes). The proportion of total Firmicutes, Bacteroides, Actinobacteria, and Proteobacteria were estimated by adding the corresponding bacterial values obtained from (i) *C*. *coccoides* group*, C. leptum* subgroup, *C. perfringens*, *Enterococcus*, *Lactobacillus* and *Staphylococcus* (total Firmicutes); (ii) *Bacteroides fragilis* group and *Prevotella* (total Bacteroides); (iii) *Bifidobacterium* and *Atopobium* cluster (total Actinobacteria); and (iv) Enterobacteriaceae (total Proteobacteria).

## Results and Discussion

Gestational age, birth mode, feeding type, birth environment, and the geographical background are known, among various other factors, as the most prominent elements shaping the neonatal gut microbiome (Penders et al., 2006; Dominguez-Bello et al., 2010; Echarri et al., 2011; Backhed et al., 2015; Bokulich et al., 2016; Tamburini et al., 2016; Hill et al., 2017). We herein aim to demonstrate the development of gut microbiota composition when these potential influences are controlled for. All 19 babies in this study are born at full-term, via vaginal delivery, and remain exclusively breast-fed until 3 to 6 months of age. Notably, all these babies also share same ethnicity (Japanese), geographical setting (Tokyo-born) as well as the birth environment (i.e., born at same clinic and spent same time, i.e., 4 days at the hospital post-birth). Hence, this highly homogenous cohort, although small in sample size, might manifest the pattern of gut microbiota development in typical natural and healthy milieus, particularly in Japanese birth population.

**FIGURE 1:**
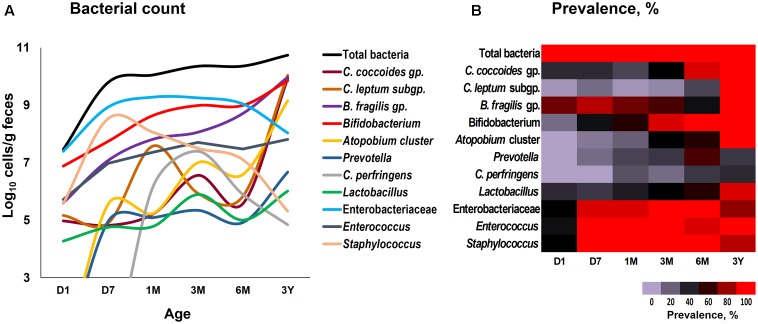
Fecal count **(A)** and prevalence **(B)** of various bacterial groups in healthy Japanese infants (*n* = 19) at different time-points during the first 3 years of life. Bacterial count is expressed as the mean of log_10_ cells/g feces. Prevalence (detection rate, %) was expressed as the percentage of infants in which the specific bacterium was detected. Age (*x*-axis): 1 day, 7 days, 1 month, 3 months, 6 months, 3 years. Numerical values on these data have been provided in Supplementary Table S1.

**FIGURE 2:**
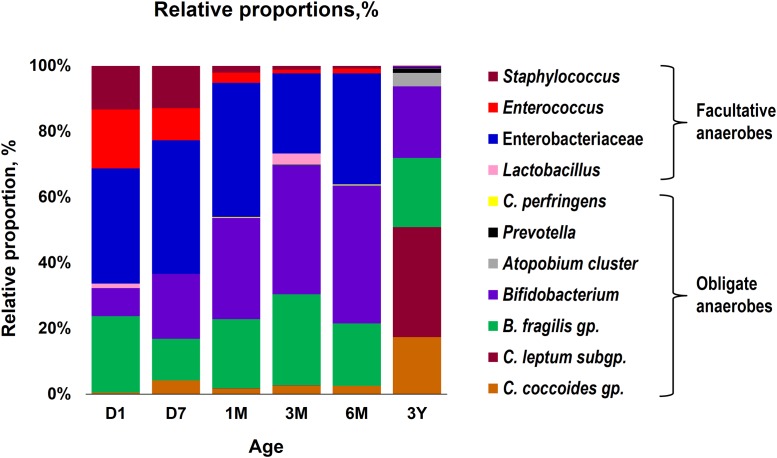
Proportional composition of the gut bacterial microbiota in healthy Japanese infants (*n* = 19) at different time-points during the first 3 years of life. Relative proportions of different bacteria were calculated by using the original arithmetical number of the bacterial count and are expressed as the percent of the total fecal bacterial count. Age (*x*-axis): 1 day, 7 days, 1 month, 3 months, 6 months, 3 years.

Our data outlining a typical infant profile, along the lines of previous reports (Aagaard et al., 2014; Ardissone et al., 2014; Collado et al., 2016; Martin et al., 2016; Chu et al., 2017; Hill et al., 2017), shows that various bacterial groups, predominantly the facultative anaerobes such as Enterobacteriaceae, *Enterococcus* and *Staphylococcus*, are already dwelling in the first intestinal discharge (i.e., meconium) of these babies (**Figure 1** and Supplementary Table S1). We noted that all (100%) of the babies harbor one or more bacteria in their meconium samples (**Figure 1B**). Since previous studies have largely used DNA-based methods, it remains debatable whether the prenatal niches encounter live bacteria or only contain dead (non-viable) bacteria that might have seeped in from maternal circulation for immune priming of the neonate. In this context, our RNA-based data of the meconium bacterial flora corroborates and fortifies the mounting body of evidence that supports the notion of *in utero* live microbial exposures (Aagaard et al., 2014; Ardissone et al., 2014; Collado et al., 2016; Chu et al., 2017), although the clear-cut routes and sources of these bacteria and their significance in context to the fetal programming and infant’s long-term health still remain largely unclear. Nevertheless, we found that at day 1, the prevalence (detection rate, %) was higher for enterobacteria, enterococci, staphylococci followed by *B. fragilis* group, bifidobacteria and lactobacilli (**Figure 1B**). Concordant with previous reports, this observation clearly suggests that the facultative anaerobes are the first settlers in the human gut whereas obligate anaerobes such as clostridia, bacteroides, and bifidobacteria thrive at later stages (Solís et al., 2010; Ardissone et al., 2014; Backhed et al., 2015; Bokulich et al., 2016; Collado et al., 2016; Hill et al., 2017). The data further demonstrate a clear predominance of these facultative anaerobes during the first few weeks of life whereafter the predominance gradually transitions toward obligate anaerobes including bifidobacteria, *B. fragilis* group, *C. coccoides* group, and *C. leptum* subgroup (**Figure 2**). At day 1, the microbiota is composed mainly of facultative anaerobes with Enterobacteriaceae (35%), enterococci (18%), and staphylococci (13%) dominating the overall composition. Among obligates, *B. fragilis* group (23%) and bifidobacteria (9%) represent the main components. However, this configuration is gradually shifted toward the predominance of obligate anaerobes, especially bifidobacteria, at the cost of facultative anaerobes viz. enterococci and staphylococci during subsequent stages. At 6 months, the microbiota is dominated by bifidobacteria (42%) followed by enterobacteria (34%) and *B. fragilis* group (19%). At 3 years, the composition is represented almost entirely (99%) by obligate anaerobes including *C. coccoides* group (17%), *C. leptum* subgroup (34%), *B. fragilis* group (21%), bifidobacteria (22%), *Atopobium* cluster (4%), and *Prevotella* (1%).

**FIGURE 3:**
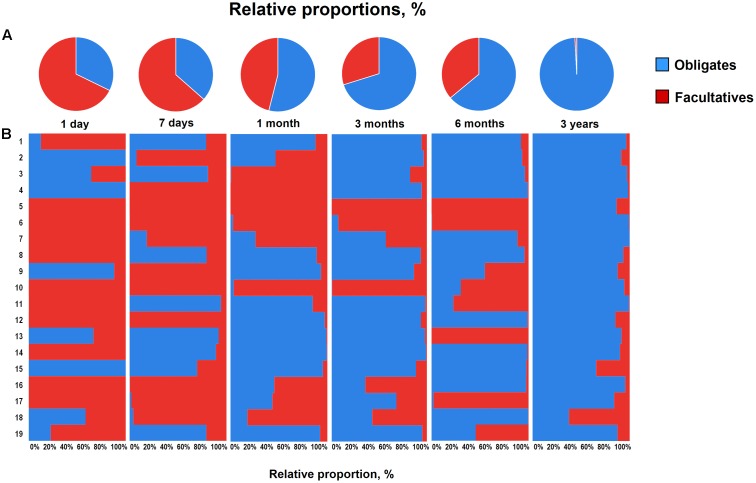
Proportional ratios of obligatory anaerobic vs. facultative anaerobic bacteria averaged **(A)** and at individual-level **(B)** in healthy Japanese infants (*n* = 19) at different time-points during the first 3 years of life. Proportions were calculated by using the original arithmetical number of the bacterial count and are expressed as the percent of the total fecal bacterial count. Obligates: *Clostridium coccoides* group, *Clostridium leptum* subgroup, *Bacteroides fragilis* group, *Bifidobacterium*, *Atopobium* cluster, *Prevotella* and *Clostridium perfringens*. Facultatives: Enterobacteriaceae, *Enterococcus*, *Lactobacillus*, and *Staphylococcus*. Age: 1 day, 7 days, 1 month, 3 months, 6 months, 3 years.

Overall, the obligate/facultative proportion is 32/68, 37/63, 54/46, 70/30, 64/36, and 99/1 at age 1 and 7 days, 1, 3, and 6 months, and 3 years, respectively (**Figure 3**). These proportions as well as the overall patterns are consistent with other studies (Arboleya et al., 2012; Backhed et al., 2015; Odamaki et al., 2016); however, interestingly, considerable variations can be seen at the individual level. For instance, several babies are dominated by obligate anaerobes even on day 1 whereas several babies are still dominated by facultative anaerobes even at 6 months of age (**Figure 3**). Interestingly, compared to full-term babies, premature babies are found to have significantly higher facultative/obligate ratio until 3 months of age or perhaps even longer (Arboleya et al., 2012); however, we noted a similar predominance of facultative anaerobes from day 1 through 6 months in several babies even in this healthy full-term cohort (**Figure 3**). Although geographical and/or methodological differences may underlie this disparity (Echarri et al., 2011), this observation of notable variations within this small cohort also clearly hints that the microbiota configuration is highly individual-specific and hence the mean values of a particular cohort might not always extrapolate the true picture of a given element or parameter. Similar to the facultative-obligate ratios, the overall mean proportions of Firmicutes, Bacteroides, Actinobacteria, and Proteobacteria communities in this cohort (Supplementary Figure S1) also seem to be consistent with previous reports (Backhed et al., 2015; Odamaki et al., 2016; Murphy et al., 2017); however, at individual-level, there are noticeable differences in the magnitude of these signatures particularly during the first 6 months. For example, at each time-point, some babies appear to be of Proteobacteria- or Actinobacteria-type while several babies show Bacteroides- or Firmicutes-predominance. Moreover, even at individual level, these signatures vary at different time-points. Nevertheless, this variation appear to diminish by age 3 years. Similar trends in inter-individual variability in the gut microbiota composition during early life have been reported in several previous studies (Roger and McCartney, 2010; Echarri et al., 2011). Interestingly, Echarri et al. (2011), while reporting considerable inter-individual variability, also observed significant differences in the gut carriage of several bacterial groups such as *Bacteroides*, staphylococci, enterobacteria in infants from two different Spanish locations just about 1000 km apart from each other. In this context, although all the infants in our study were born at the same clinic, the effect of differences in their residential locations (e.g., different wards and cities of Tokyo) on the gut microbiota composition cannot be ruled out completely. In a recent study on children from different Asian countries, our colleagues clustered the gut microbiome signatures into two groups, P-type (predominated by *Prevotella*) and BB-type (predominated by *Bifidobacterium* and *Bacteroides*), and reported that Japanese children are mostly of BB-type (Nakayama et al., 2015). In the present study, the data at 3 years seem to concord with this notion because many babies are dominated by Actinobacteria and Bacteroides and hence might arbitrarily be envisaged to be of BB-type (Supplementary Figure S1). The overall composition at 3 years – wherein *B. fragilis* group and *Bifidobacterium* together make up for about 43% of the total fecal bacterial count – also seem to corroborate this notion (**Figure 2**). Nevertheless, we notice slight variation in these arrays even at 3 years of age since some babies are still dominated by Firmicutes community (Supplementary Figure S1). This disparity with the previous report by Nakayama et al. (2015) may be due to the methodological (RT-qPCR vs. pyrosequencing) or cohort-age (3 years vs. 7–11 years) differences; or maybe the microbiota is still under transition state at age 3 years and hence attains an adult-like configuration at a later age than previously suspected. Nonetheless, the data do hint that even at 3 years of age, there are considerable individual-level differences in the microbiota composition within this small but controlled cohort.

**FIGURE 4:**
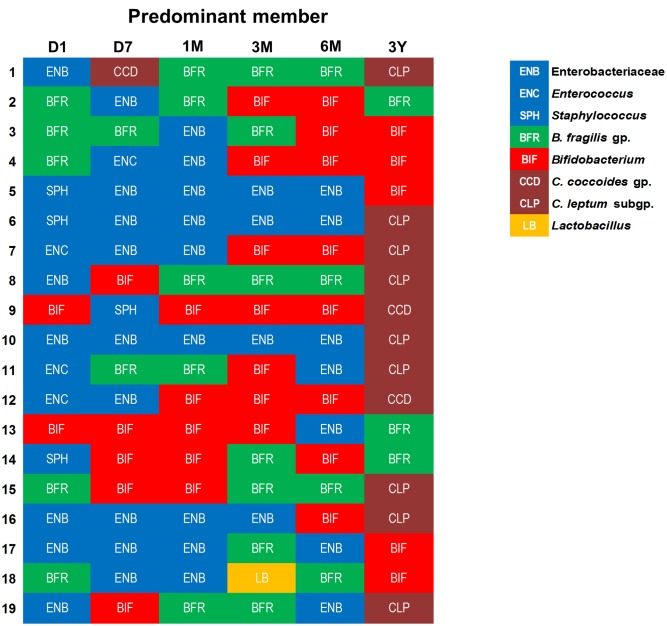
An arbitrary overview of the bacterial predominance in healthy Japanese infants (*n* = 19) at different time-points during the first 3 years of life. The abundance was estimated based on the fecal bacterial counts (log_10_ cells/g feces) wherein (out of various bacteria analyzed in this study) the bacterial clade with highest fecal count at a given time-point was considered as the most abundant clade among all the targeted bacterial groups. Age: 1 day, 7 days, 1 month, 3 months, 6 months, 3 years.

Similar individual-specific differences were also reflected when we estimated the bacterial predominance by evaluating the counts of different bacterial groups and tagging the most abundant clade, i.e., the one with highest bacterial count at a given time-point (**Figure 4**). We noted that at first day of life, the microbiota is dominated mainly by Enterobacteriaceae, staphylococci, or enterococci, or by *B. fragilis* group in some babies, but this dominance gradually shifts toward *B. fragilis* group and bifidobacteria during subsequent phases. Finally, by age 3 years, the predominance switches completely toward obligate anaerobes, possibly in conjunction with the natural process of transition toward adult-microbiota configuration. Weaning, i.e., the introduction (and type) of solid foods, may also be one of the key factors driving this compositional shift between 6 months and 3 years. Nevertheless, even at 3 years, the spectrum of this predominance is diverse with most babies being dominated by *C. coccoides* group or *C. leptum* subgroup whereas few babies are dominated by *B. fragilis* group or bifidobacteria. This observation of considerable inter-individual disparity even within this healthy homogenous cohort underpins the importance of assessing not only the birth mode, feeding type, gestational age and ethnic/geographical background but also other potential factors (which we were unfortunately not able to peruse due to information paucity) such as maternal microbiome and gestational diet, breast-milk’s microbial/biochemical composition, prior drug exposures, household environment, host genetics, intestinal physiology and hormonal status etc. when investigating the elements that shape and drive the early life gut microbiota dynamics (Echarri et al., 2011; Koenig et al., 2011; Yatsunenko et al., 2012; Blekhman et al., 2015; Bokulich et al., 2016; Charbonneau et al., 2016; Chu et al., 2016, 2017; Matsuki et al., 2016).

This small study has several strengths and limitations. The main strength is the homogenous nature of the cohort controlled for gestational age, birth mode, feeding type, place of birth, ethnic and geographical background etc. Also, all the babies remained apparently healthy (without any serious illness or considerable exposure to antibiotics/drugs) during the study period. In addition, the cohort was also quite homogenous in terms of the babies’ weight during different time-points (**Table 1**), which actually did not allow us to statistically examine the correlation if any of gut microbiota with host bodyweight. The main limitation of the study is the lack of data on multiple outstanding elements (e.g., maternal microbiome and diet during pregnancy, maternal disease history and drug exposures, familial/household environment, siblings, pets, etc.) that may influence the gut microbiota development. Small samples size is another limitation that merits validation of similar analyses in further larger cohort studies. Another limitation is that the bacteria were quantified by RT-qPCR assays using specific primer sets for major gut bacterial groups and genera and hence the microbiota data presented in here may not truly represent the whole array of the gut microbiota; although we have previously validated that the sum of the fecal counts of all the bacterial groups enumerated herein using RT-qPCR assays correspond to 71.3 ± 9.4% (mean ± SD) of total intestinal bacterial count as estimated by *in situ* hybridization using a universal probe (Hasegawa et al., 2015). Nevertheless, as mentioned beforehand, this quantitative approach could also be considered as a strength of this study, especially considering the high detection sensitivity of these assays and the lack of availability of such numeric and longitudinal data on major human gut bacterial clades.

## Conclusion

In summary, this addendum demonstrates a quantitative bird’s-eye view of the development of the gut microbiota community composition during the first 3 years of life in typically natural and healthy milieus, i.e., full-term, vaginal-birth, and exclusive-breast-feeding (and same geographical setting). The findings from this small but homogenous cohort of babies continue to expand our understanding of the maturation of human intestinal microbiota during neonatal and early childhood period, a phase where the microbiota is in most fluctuating stages of its lifespan. The data provide important numeric information about the population levels of various important dominant and subdominant gut bacterial groups inhabiting the early infant gut and hence should prove to be informative and facilitative for future studies exploring various aspects of the human gut microbiota. The data illustrate how the gut microbiota configuration evolves during early life and hint that there are certainly many more known or yet-to-be-known intrinsic and extrinsic factors (other than the gestation age, birth-mode, feeding type, place of birth and geographical background) that may impact the early-life gut microbiome even in healthy infants and hence may underlie the inter-individual disparities in the microbiota composition. Studies deciphering these inter-individual dynamics and elements of the gut microbiota composition would certainly offer new hypotheses, insights and avenues for improving the infant’s intestinal as well as overall long-term health while refining our understanding of this complex gut microbial ecosystem.

## Author Contributions

RN, HT, and YY: conceived and designed the study; RN and HT: performed the experiments; RN: analyzed the data; HT and KK: coordinated the sample collection; RN: wrote the manuscript; HT, TT, KN, and YY: checked and revised the manuscript; RN, HT, TT, KN, KK, SN, and YY: approved the final version of manuscript.

## Conflict of Interest Statement

The authors declare that the research was conducted in the absence of any commercial or financial relationships that could be construed as a potential conflict of interest.
