# Supplementary material for: Ontogenesis of the Gut Microbiota Composition in Healthy, Full-Term, Vaginally Born and Breast-Fed Infants over the First 3 Years of Life: A Quantitative Bird’s-Eye View
Source: Front Microbiol. 2017 Jul 21;8:1388. doi: 10.3389/fmicb.2017.01388 (PMC5519616; doi:10.3389/fmicb.2017.01388)
Supplement: Supplementary file 1 [file Table_1.PDF]

Manuscript title: Ontogenesis of the gut microbiota composition in healthy, full-term, vaginally-born and breast-fed infants over the first 3 years of life: a quantitative bird's-eye view

Authors: Ravinder Nagpal, Hirokazu Tsuji, Takuya Takahashi, Koji Nomoto, Kazunari Kawashima, Satoru Nagata, Yuichiro Yamashiro

Supplementary Table S1. Fecal count (log_{10} cells/ g feces) and prevalence (detection rate, %) of various bacterial groups at different time-points during the first 3 years of life. Total bacteria are expressed as the sum of all the other bacteria listed in the table. Prevalence (detection rate, %) was expressed as the percentage of infants in which the specific bacterium was detected. ND: not detected.

	Log_{10} cells/g feces, Mean ± SD [Prevalence, %]					
	Age					
	1 day	7 days	1 month	3 months	6 months	3 years
Total bacteria	7.5 ± 2.2 [100]	9.8 ± 0.5 [100]	10.1 ± 0.4 [100]	10.4 ± 0.3 [100]	10.4 ± 0.7 [100]	10.7 ± 0.2 [100]
C. coccoides group	5.0 ± 1.7 [47]	4.8 ± 2.3 [47]	5.2 ± 3.0 [37]	6.6 ± 2.4 [63]	5.6 ± 2.3 [95]	9.9 ± 0.2 [100]
C. leptum subgroup	5.2 [5]	4.9 ± 1.4 [21]	7.6 [5]	5.9 ± 3.4 [11]	5.8 ± 1.0 [37]	10.0 ± 0.7 [100]
B. fragilis group	5.6 ± 2.0 [79]	7.1 ± 2.1 [90]	7.8 ± 2.5 [79]	8.1 ± 2.9 [74]	8.7 ± 2.5 [58]	10.0 ± 0.4 [100]
Bifidobacterium	6.9 ± 2.4 [21]	7.7 ± 2.1 [58]	8.6 ± 2.0 [68]	9.0 ± 1.9 [95]	9.0 ± 1.8 [100]	9.8 ± 0.5 [100]
Atopobium cluster	ND ---	5.6 ± 0.9 [16]	5.2 ± 0.7 [26]	7.0 ± 0.9 [63]	6.6 ± 1.4 [68]	9.2 ± 0.5 [100]
Prevotella	ND ---	5.0 ± 0.2 [21]	5.1 ± 0.6 [37]	5.3 ± 0.7 [42]	4.9 ± 0.6 [74]	6.7 ± 1.4 [42]
C. perfringens	ND ---	ND ---	6.2 ± 1.4 [32]	7.4 ± 0.6 [21]	5.9 ± 1.5 [42]	4.8 ± 1.0 [47]
Lactobacillus	4.3 ± 0.8 [47]	4.8 ± 1.1 [42]	4.8 ± 1.6 [53]	5.9 ± 2.7 [63]	5.0 ± 2.3 [68]	6.0 ± 1.6 [95]
Enterobacteriaceae	7.4 ± 2.1 [63]	8.9 ± 1.5 [95]	9.3 ± 0.9 [95]	9.3 ± 0.6 [100]	9.0 ± 1.4 [100]	8.0 ± 0.6 [84]
Enterococcus	5.7 ± 2.5 [58]	7.0 ± 2.1 [100]	7.4 ± 1.7 [100]	7.7 ± 0.9 [100]	7.5 ± 1.2 [95]	7.8 ± 0.7 [100]
Staphylococcus	5.6 ± 1.8 [63]	8.5 ± 0.5 [100]	8.1 ± 0.5 [100]	7.5 ± 0.9 [100]	7.1 ± 1.0 [100]	5.3 ± 0.8 [90]
