# Supplementary material for: Ontogenesis of the Gut Microbiota Composition in Healthy, Full-Term, Vaginally Born and Breast-Fed Infants over the First 3 Years of Life: A Quantitative Bird’s-Eye View
Source: Front Microbiol. 2017 Jul 21;8:1388. doi: 10.3389/fmicb.2017.01388 (PMC5519616; doi:10.3389/fmicb.2017.01388)

Manuscript title: Ontogenesis of the gut microbiota composition in healthy, full-term, vaginally-born and breast-fed infants over the first 3 years of life: a quantitative bird's-eye view

Authors: Ravinder Nagpal, Hirokazu Tsuji, Takuya Takahashi, Koji Nomoto, Kazunari Kawashima, Satoru Nagata, Yuichiro Yamashiro

Supplementary Fig. S1. Proportional ratios of total Firmicutes, Bacteroides, Actinobacteria and Proteobacteria averaged (A) and at individual-level (B) in healthy Japanese infants (n=19) at different time-points during the first 3 years of life. Proportions were calculated by using the original arithmetical number of the bacterial count and are expressed as the percent of the total fecal bacterial count. Firmicutes: *C. coccoides* group, *C. leptum* subgroup, *Clostridium perfringens*, *Enterococcus*, *Lactobacillus* and *Staphylococcus*. Bacteroides: *Bacteroides fragilis* group and *Prevotella*. Actinobacteria: *Bifidobacterium* genus and *Atopobium* cluster. Proteobacteria: Enterobacteriaceae.

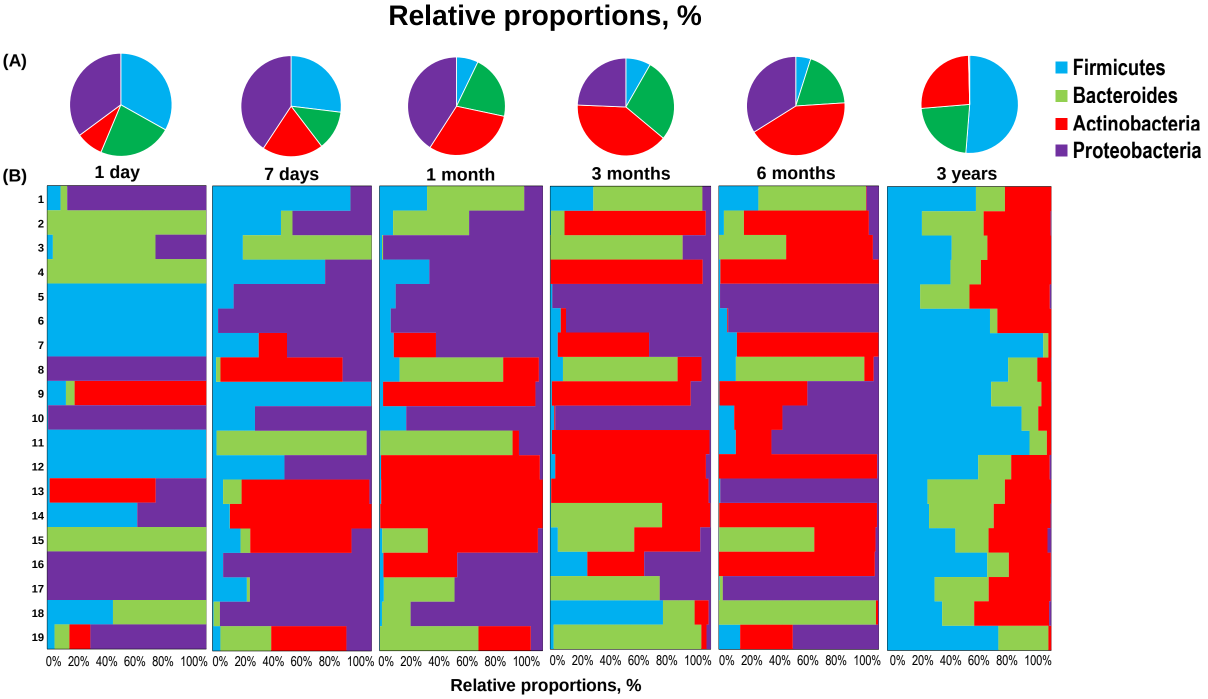

Supplement: Supplementary file 2 [file Image_1.pdf]
